# Manufacturing and Application of 3D Printed Photo Fenton Reactors for Wastewater Treatment

**DOI:** 10.3390/ijerph18094885

**Published:** 2021-05-04

**Authors:** Kourosh Nasr Esfahani, Mohammad Damous Zandi, J. Antonio Travieso-Rodriguez, Moisès Graells, Montserrat Pérez-Moya

**Affiliations:** 1 Chemical Engineering Department, Campus Diagonal-Besòs, Universitat Politècnica de Catalunya, Av. Eduard Maristany, 16, 08019 Barcelona, Spain; kourosh.nasr.esfahani@upc.edu (K.N.E.); moises.graells@upc.edu (M.G.); 2 Mechanical Engineering Department, Campus Diagonal-Besòs, Universitat Politècnica de Catalunya, Av. Eduard Maristany, 16, 08019 Barcelona, Spain; mohammad.damous.zandi@upc.edu (M.D.Z.); antonio.travieso@upc.edu (J.A.T.-R.)

**Keywords:** photo-Fenton, wastewater treatment, PLA, Timberfill^®^, 3D printing, raceway pond

## Abstract

Additive manufacturing (AM) or 3D printing offers a new paradigm for designing and developing chemical reactors, in particular, prototypes. The use of 3D printers has been increasing, their performance has been improving, and their price has been reducing. While the general trend is clear, particular applications need to be assessed for their practicality. This study develops and follows a systematic approach to the prototyping of Advanced Oxidation Processes (AOP) reactors. Specifically, this work evaluates and discusses different printable materials in terms of mechanical and chemical resistance to photo-Fenton reactants. Metallic and ceramic materials are shown to be impracticable due to their high printing cost. Polymeric and composite materials are sieved according to criteria such as biodegradability, chemical, thermal, and mechanical resistance. Finally, 3D-printed prototypes are produced and tested in terms of leakage and resistance to the photo-Fenton reacting environment. Polylactic acid (PLA) and wood–PLA composite (Timberfill^®^) were selected, and lab-scale raceway pond reactors (RPR) were printed accordingly. They were next exposed to $H_{2}O_{2}$/Fe(II) solutions at pH = 3 ± 0.2 and UV radiation. After 48 h reaction tests, results revealed that the Timberfill^®^ reactor produced higher Total Organic Carbon (TOC) concentrations (9.6 mg·L^−1^) than that obtained for the PLA reactor (5.5 mg·L^−1^) and Pyrex^®^ reactor (5.2 mg·L^−1^), which suggests the interference of Timberfill^®^ with the reaction. The work also considers and discusses further chemical and mechanical criteria that also favor PLA for 3D-printing Fenton and photo-Fenton reactors. Finally, the work also provides a detailed explanation of the printing parameters used and guidelines for preparing prototypes.

## 1. Introduction

Computer-aided design (CAD) and computer-aided manufacturing (CAM) technologies are today essential for designing and manufacturing functional objects. Additive manufacturing (AM) or 3D printing takes advantage of digitalization to enable crafting unique pieces at a cost and speed comparable to that given by mass production [1]. Although still in an early stage, the utilization of AM for producing chemical reactors is emerging as an opportunity to explore new geometries and integrated designs and speed up their validation. Three-dimensional (3D) printing is an enabling technology that is making the shaping of complex reactor prototypes increasingly cheaper and faster. Such rapid prototyping will shorten the development process and the testing of inexpensive and more sustainable materials, which ultimately will lead to better processes.

Some works have reviewed the application of AM in chemistry and pharmaceutical, biological, and chemical engineering [2,3,4]. Most of the recent works on this area report on approaches converging to process intensification via the miniaturization of the equipment and the adoption of continuous processing (continuous flow chemistry [5,6], milli-, and micro-fluidics [7,8]) and to modularity and on-demand reactionware [9,10]. Three-dimensional (3D) printing has also been explored for wastewater treatment processes; most developments reported aim again at process intensification through the design and production of high-surface-area biocarriers that enhance the performance of moving bed biofilm reactors (MBBR) [11,12,13].

However, the application of 3D printing and computational modeling (CAD, CAM) for the design and fabrication of chemical reactors for advanced oxidation processes (AOPs), in particular photo-Fenton reactors, has been hardly addressed [14,15], and a systematic approach for material selection for 3D printing such reactors is also missing [1].

Fenton-based processes are very successful options in terms of effective pollutant degradation that can be boosted by different ways such as solar light [16] or electrochemical process [17]. The photo-Fenton reaction can be summarized as a catalytic reaction of ferrous iron (${\mathrm{Fe}}^{2+}$) reagent with hydrogen peroxide in an acidic or circumneutral pH yielding hydroxyl radicals. The presence of UV-VIS radiation enhances the reaction rate; therefore, this process is strongly dependent on iron concentration and irradiance, which are important factors in reactor design and process operation [18]. Iron nanoparticles present higher surface energy than iron salts, and they have also been proposed and tested to substitute iron sulfate and reduce the sludge produced in the reactor [19,20,21]. In any case, photoreactor prototyping and subsequent testing are crucial for the modeling and application of the photo-Fenton processes.

Although the application of AM offers significant opportunities in controlling fluid dynamics and optimized reactor geometries, it poses new problems regarding the properties of the materials used for 3D printing, which are often only discussed in the context of mechanical characteristics, and rarely because of their chemical functionality [3]. Indeed, materials selection for 3D printing needs to be considered according to chemical constraints such as solvent compatibility with the various reagents [22].

This paper addresses the selection of materials according to different criteria for reactor prototyping and subsequent testing of the chemical suitability of the reactor for carrying out advanced oxidation processes, in particular, photo-Fenton processes, and it proposes a systematic approach to the selection procedure.

## 2. Materials and Methods

**Figure 1:**
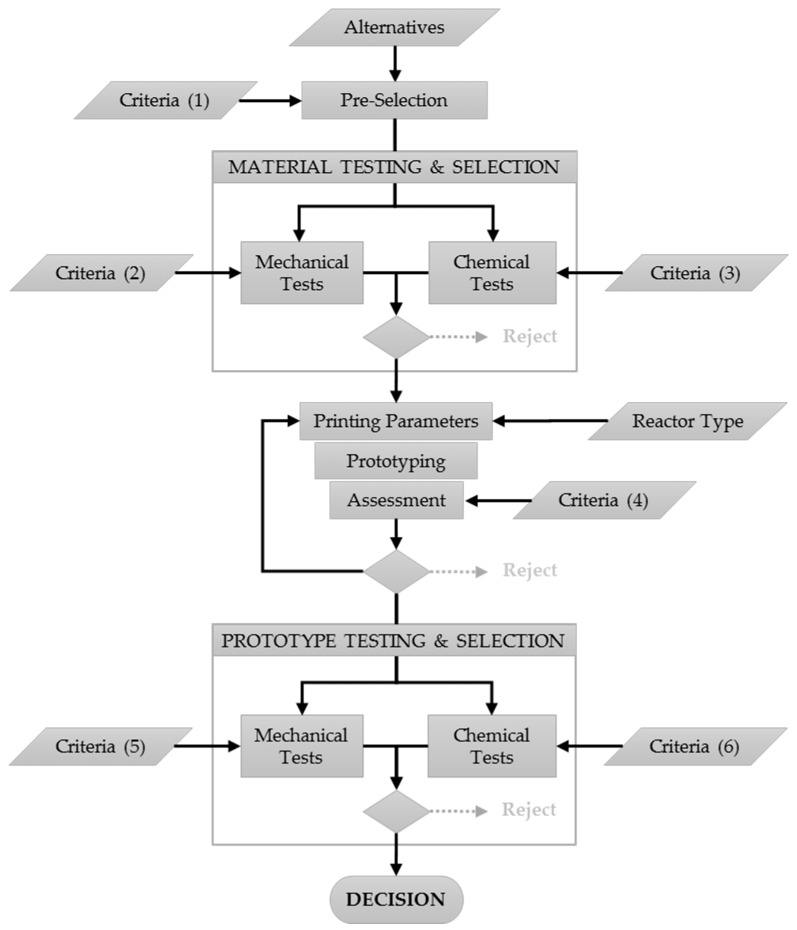
Proposed methodology: tests and criteria for preparing 3D-printed photo-Fenton reactors.

The process proposed for determining a suitable material satisfying both chemical and mechanical requirements is illustrated in Figure 1. The diagram shows multiple criteria in several steps.

### 2.1. Materials

The printing filaments used in the reactor manufacture were supplied by the Filamentum Company (Hulín, Czech Republic). Concerning the considerable effect of the material characterization including deformability, safety, printability, and stiffness or bending, the manufacture’s material datasheets were considered, and also some tests were performed. Those tests will be described in the following subsections. The printed parts have been manufactured using a Sigma printer by the BCN3D Company (Barcelona, Spain) and fused filament fabrication (FFF).

Hydrogen peroxide ($H_{2}O_{2}$) 33% *w/v* in analytical-grade was supplied from Panreac Química SLU (Barcelona, Spain). Iron sulfate heptahydrate (${\mathrm{FeS}}_{4}$_7H_2_O) by Sigma-Aldrich (St. Louis, MO, USA) was used as another Fenton reagent. Sulfuric acid H_2_SO_4_ (95–97%) for adjusting the initial pH and pure caffeine as the contaminant were purchased from Sigma-Aldrich (St. Louis, MO, USA). All solutions were prepared with deionized water having conductivity lower than 1.25 µs provided by Adesco S.A. (Barcelona, Spain). Actinic BL TL-DK 10 W/10 1SL lamp (UVA-UVB) (Barcelona LED, Barcelona, Spain) with the main emission at 254 nm was used as the UV light source.

### 2.2. Lab-Scale Experiments and Analytical Procedures

The design of experiments is summarized in three main parts:

- A set of primary tests with different materials is carried out to determine the chemical behavior of the pieces of the material before and after the printing process. Criteria #3, Section 3.2.2 Chemical Tests.
- Once the reactor prototypes are printed using each of the selected materials, their viability as a Fenton reactor is assessed. Thus, the same reactions are carried out without UV radiation in the reactor prototypes and, parallelly, in a Pyrex^®^ flask used as a blank test. Criteria #6, Section 3.4.2 Chemical Tests.
- Finally, the same assays are repeated for testing the reactor prototypes under the photo-Fenton environment. This time, the assays are performed under UV irradiation and with caffeine as a contaminant. Caffeine is selected as a convenient substance for these new assays since, besides its easy availability and manageability, it is considered as an emerging contaminant (mostly due to its high water solubility and low degradability) that has been widely studied in the literature [23,24,25,26]. Criteria #6, Section 3.4.2 Chemical Tests.

All reagents were added at the beginning of the assays. The temperature was not controlled. Samples were taken every 20.0 min and were refrigerated after extraction to slow down any further degradation of the organic matter.

### 2.3. Procedures and Equipment

The concentration of total organic carbon was measured by Shimadzu TOC-VCSH/CSN analyzer, Japan. $H_{2}O_{2}$ concentration was determined through the spectrophotometric method developed by Nogueira et al. [27]. The absorption at 450 nm was detected via a Lambda 365 UV/Vis spectrophotometer (Perkin Elmer, Ohio, OH, USA).

The thermogravimetric analysis (TGA) of the samples was carried out by TGA Q50 PerkinElmer. For the tensile tests, the universal test machine Microtest has been used equipped with a 25 kN load cell, 50 mm extensometer, Spider and Microtest data acquisition system.

Important tests such as elasticity, tension, and elongation were performed following the ASTM D638-14 standard [28].

## 3. Results

### 3.1. Pre-Selection of Alternative Printing Materials

A wide range of 3D printing materials with different characteristics are available, whose properties and durability can be altered by the environment set by the photo-Fenton process [3].

The most common groups of materials used for 3D printing [29] are presented in Table 1.

Ceramic materials have been studied and recommended for applications requiring high corrosion resistance [30]. Metals have well-known properties and high mechanical resistance. However, additive manufacturing is complex and expensive for both groups of materials. On the other hand, polymers and polymer-based composites resist corrosion (to a lesser extent), their mechanical strength is acceptable (enough to tolerate the weight of the liquid inside the reactor), they are much lighter, and their manufacturing cost is much lower. They can be manufactured through both industrial and domestic additive manufacturing techniques, such as Fused Filament Fabrication (FFF). These considerations, supported by some studies published in the bibliography [29], allow a first broad selection, which is shown in Table 2.

Currently, there are many different polymeric and polymeric-based composite materials available on the market that can be easily employed for FFF. Some of the most common are Nylon [22], Polycarbonate (PC) [31], Polyvinyl alcohol (PVA), Acrylonitrile Butadiene Styrene (ABS), Polylactic acid (PLA), and a variety of composites such as Timberfill^®^, a PLA-based material with wood fibers manufactured by Fillamentum Manufacturing Czech s.r.o [32].

While these materials share important features as members of the same class, there are important differences regarding the specific application they are intended for. Hence, they can be preliminarily filtered using general criteria and easily available information (non-standardized data, commercial datasheets [33], etc.), which allows avoiding more expensive testing.

Thus, Nylon is a non-biodegradable material, with a very high cost, compared to other filaments, and with low heat resistance. These are the reasons considered to reject it. PC is a thermoplastic polymer. Normally, it is a strong and tough material that can be easily worked, molded, and thermoformed [34]. The cost is higher than other thermoplastics in the market, which is a serious drawback. PVA can be readily rejected due to its distinctive solubility in water, which is detrimental to its use in aqueous media. Incidentally, PVA is mainly used in FFF to build supporting structures upon which the true piece is printed; once finished, PVA can be easily eliminated due to its water solubility [33].

On the other hand, ABS, PLA, and Timberfill^®^ can be preliminarily, selected since they do not present any of these major drawbacks.

ABS is one of the most widely used thermoplastics. It could be used in many industrial applications such as automotive components. Among its valuable properties, toughness and good resistance to high temperatures are remarkable [35]. In addition, the price is very competitive compared to most materials available in the market.

PLA is a biodegradable material obtained from renewable sources such as cornstarch, etc. Thus, it is the most environmentally friendly material among many other 3D printing materials. Its advantages are small thermal contractility, very good mechanical resistance, and low price [35].

An alternate biodegradable material proposed is Timberfill^®^, which results from PLA combined with wood fibers. Although designed for a purely aesthetic purpose (imitating objects with a wood aspect), it is worth analyzing due to its reasonable price. Its inconvenience is that its mechanical strength is lower than that of PLA [36].

All this is summarized in Table 3.

### 3.2. Material Testing and Selection

After the pre-selection (screening phase) of ABS, PLA, and Timberfill^®^ as promising materials for 3D printing of Fenton and photo Fenton reactors, the performance of these materials needs to be further examined and compared based on chemical and mechanical criteria before attempting the printing process.

#### 3.2.1. Mechanical Tests

The mechanical properties required could be determined using accepted standard measurement and assays, which define the material behavior with respect to its resistance to load. Large strain plasticity, as observed in various materials, is one of the well-known phenomena requiring combined geometric and material nonlinear analysis of solids [37,38]. Additionally, each one of these responses may be defined in relation to the loading mode: tension, compression, flexure, shear, or torsion [39], which depends on the way that the component carries out the loads. Regarding the practicability of 3D printing a reactor, static tests such as tensile and flexural tests have been executed on the mentioned materials.

In general, the materials selected for a 3D printing reactor require certain mechanical criteria to be verified. In particular, three mechanical criteria have been considered. In the following sections, the conditions that each evaluated material needs to meet with each of the three criteria considered will be explained.

##### Criterion 2.1: Printability

To manufacture the reactors, it is necessary to find a material that is easy to print. This means that the selected materials could be printed without presenting the most common problems that occur in an FFF process, such as Extruder plugging [40], the first layer of material does not stick to the hot base [41], less or more material extruded during the process [42], overheating [43], wrapping (very common in ABS) [44], uneven printing [45], etc.

Table 4 shows a preliminary evaluation according to printable conditions (Criterion 2.1), for PLA, Timberfill^®^, and ABS. The characteristic properties required for this assessment can be extracted from the materials datasheets and do not require performing further assays. The material has been considered to meet this criterion if it does not present any of the problems mentioned in the previous paragraph or if they can be solved otherwise. Hence, not all materials meet this criterion in the same way, as shown in Table 4, although none of them can be discarded on this basis.

##### Criterion 2.2: Stiffness

Stiffness is another significant aspect to be considered. Stiffness is the extent to which an object resists deformation in response to an applied force [46]. Regardless of whether stiffness is closely related to the internal infill of the pieces, the properties of the selected material also have an important influence on it.

The datasheets by the material’s manufacturers provide enough information to assess stiffness. To consider that a material meets this criterion, a limit of 53 MPa has been established, which allows discarding ABS on this basis (Table 4, Criterion #2.2).

##### Criterion 2.3: Heated Bed

The hotbed is the element that is responsible for heating the base of the 3D printer so that the parts remain adhered to during the manufacturing process. This avoids or reduces the effect of wrapping (pieces that detach and bend during the printing process). Not all materials require a hotbed printer to be printed [41].

Whether a hotbed is required or not can be also extracted from the manufacturer’s datasheets for the three pre-selected materials (Table 4, Criterion #2.3). If the hotbed is needed, the material is considered not to meet this criterion, which is the case of ABS.

Table 4 summarizes criteria #2 and the decisions made accordingly. Printability is better in PLA and ABS than Timberfill^®^. The last one is printed with greater difficulty than the previous ones, but by increasing the extruder diameter and decreasing the layer height [47], the problem is solved. The stiffness in the case of ABS decreased compared to PLA and Timberfill^®^ [48]. Print using a heated bed is only necessary in the case of ABS. So, the partial conclusion according to criteria #2 is that PLA and Timberfill^®^ are more appropriate materials and ABS can be discarded.

#### 3.2.2. Chemical Tests

A wide assortment of polymers resisting high temperatures and chemicals is currently available. Apart from printable metals (composites, powders, alloys) and ceramics, different types of inert polymers have been studied for 3D printing reactors: fluoropolymers [49], Acrylonitrile butadiene styrene (ABS) [50]; Polycarbonate (PC); Nylon [31]; Polypropylene (PP) [51], Polystyrene (PS) [52]; and Polylactic acid (PLA) [53]. While the first polymers used in 3D printing had limited chemical and thermal stability, new materials have been introduced with increased performance [54].

Thus, materials commonly used for AM have been tested and ranked accordingly, for instance regarding the effect of prolonged exposure to an aggressive organic solvent such as methylene chloride [22].

General ideas for material selection and reactor design and development can be found in the literature [3]. However, to the best knowledge of the authors, no studies address the specific issues of Fenton and photo-Fenton processes.

For the Fenton and photo-Fenton reactions, tests should be designed and performed to assess the chemical stress that the reacting media causes to the vessel holding it. The thermal stability of the material under heat stress and radiation should be considered as well.

Thus, three criteria can be envisaged according to the resistance to the Fenton reactants and intermediates (as the high oxidant hydroxyl radicals), the resistance to the irradiation itself causing the photo-Fenton process, and the resistance to the temperature changes that the reactor may experience.

Since the chemical stress produced by the photo-Fenton reaction is intrinsically linked to the stress produced by irradiation, both effects will be jointly tested after they are described (Criteria 1 and 2). Certainly, the irradiation stress could be tested separately from the Fenton reaction to discern the individual effects, but they are simultaneously addressed for the practical purposes of material selection.

##### Criterion 3.1: Reaction Environment

The presence of the Fenton reactants, the need for acidic media, and the production of highly oxidant species are the conditions that should be specifically considered. Furthermore, since the purpose of Fenton and photo-Fenton treatments is to remove organic contaminants from water, a reaction media attacking a polymeric reactor not only threatens the consistency of the reactor but also, and more significantly, the quality of the water and its measurement. The measurement of organic matter is commonly performed via a lumped parameter such as TOC [55], which also poses a detection limit and a practical threshold for the amount of polymeric material that could be detected in a water sample.

According to the literature [56,57], a wide range of Fenton reactants ratios ($H_{2}O_{2}$:Fe(II) from 5:1 to 100:1) have been applied for treating different contaminants in wastewaters. Hence, a test has been designed by establishing 300 ± 10 mg·L^−1^ $H_{2}O_{2}$ and 10 ± 0.1 mg·L^−1^ Fe(II), which sets the Fenton reagent mass ratio to 30:1. The ratio selected is within the threshold previously commented, the iron concentration is the maximum value allowed in wastewaters in Spain [58], and the hydrogen peroxide concentration doubles that used in recent works [59]. The media, a volume of 500 mL, is also set at the usual reaction pH, 3 ± 0.2.

A sample of each material to be tested is prepared in pieces of 80 × 10 × 4 mm (approximately, which corresponds to about 3 gr mass). The selected materials are dipped in the synthetic water prepared for 48 h, which is considered long enough for a first material screening. The same procedure is carried out for a control sample, which is dipped in distilled water. The initial TOC concentration is measured for all the assays (test and control).

To pass Criterion 3.1, TOC concentration after 48 h should not increase the initial TOC concentration beyond an acceptable measurement limit to discard the hypothesis of a migration of the building material to the reaction environment. This limit is set to 0.23 mg·L^−1^, according to the precision of the TOC analyzer [60]. In addition, the aspect of the material should not present any modification at sight.

##### Criterion 3.2: UV Resistance

Common synthetic polymers can be attacked by sunlight, and the photo-Fenton process requires the presence of UV-vis light [55]. The literature reports that the presence of UV-vis light (λ ≤ 580 nm) allows reducing Fe (III) again into Fe (II), which in turn produces further ^•^OH radicals and results in a cycle continuously supplying ^•^OH until $H_{2}O_{2}$ is depleted. Shorter wavelengths (λ ≤ 310 nm) cause hydrogen peroxide photolysis and the direct production of extra ^•^OH. Therefore, the oxidation rate of photo-Fenton is much higher than that of the Fenton process.

Thus, materials should be tested under such irradiating conditions. UV light (artificial and solar light) was used and the conditions to pass Criterion 2.2 are the same set for Criterion 2.1 after 48 h.

ABS is reported to significantly downgrade [50,61] when exposed to UV radiation in an oxygen-rich environment. While this reinforces the rejection of ABS as inappropriate for the photo-Fenton reaction, it also suggests that the suitability for the Fenton reaction should be further investigated if required. Hence, tests were produced for Timberfill^®^ and PLA and no significant changes in TOC and pH were measured after 48 h. The results are summarized in Table 5.

##### Criterion 3.3: Thermal Stability

Thermal degradation is another aspect to be considered [62]. When temperature exceeds the glass transition temperature of the polymer, the materials, as well as their products would become easy to distort, wrinkle, or tear, and the mechanical properties would fall sharply [63]. The exothermic photo-Fenton process under irradiation experiences an increase in temperature; this may cause additional stress.

Positively, higher temperatures could lead to a more efficient use of $H_{2}O_{2}$ (amount of TOC removed per amount of $H_{2}O_{2}$ used), since temperature may increase the generation of ^•^OH radicals at low Fe^2+^ concentration. However, increasing the operating temperature as a way of improving the Fenton process has been scarcely investigated because the idea of thermal decomposition of $H_{2}O_{2}$ into $O_{2}$ and $H_{2}O$, seems to be widely accepted as a serious drawback [64]. Therefore, the common temperature range in Fenton and photo Fenton processes may include between ambient temperature and 45–50 °C [65].

Thermogravimetric analysis (TGA) has proved to be a suitable technique to study the thermal stability of polymeric materials, and it is proposed to assess the thermal stability of candidate materials. The weight of a sample is measured as the temperature changes, and the measured changes are associated with decomposition and oxidation reactions as well as physical processes such as sublimation, vaporization, and desorption, which provide valuable information on materials selection [66].

**Figure 2:**
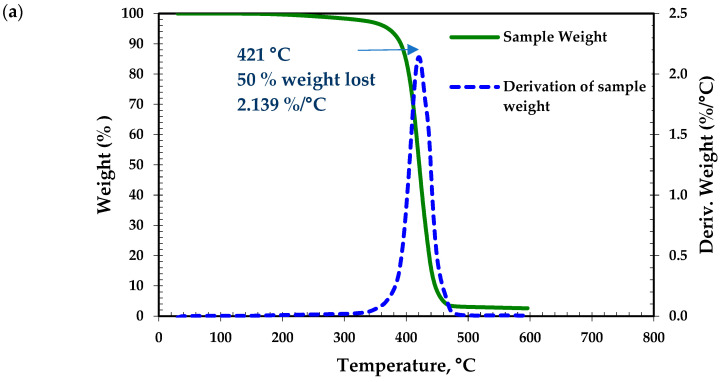

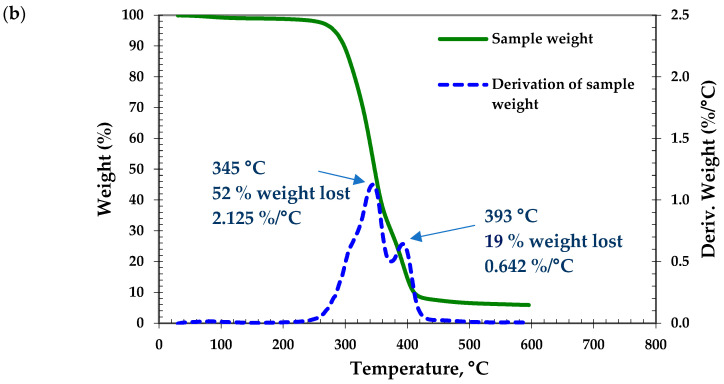
Thermogravimetric analysis (TGA) of PLA (**a**) and Timberfill^®^ (**b**).

ABS, discarded by previous criteria, is reported to have low thermal stability [67], which supports the decision. For PLA and Timberfill^®^, assays were made to measure their thermal stability. A criterion is set so that the material should present a maximum thermal decomposition rate at temperatures beyond 300 °C. Results in Figure 2 show a maximum thermal decomposition rate for PLA at 421 °C and a couple of peaks for Timberfill^®^, both beyond 300 °C. The two peaks can be explained by the fact that Timberfill^®^ is a mixture (wood and PLA).

Table 5 summarizes the criteria and the decisions derived from them corresponding to the comparison investigation carried out in this section, according to the thermal stability, too.

### 3.3. Reactor Type, Prototyping, Printing Parameters

Selecting the reactor type (geometry, optimization, etc.) is a process out of the scope of this work. Despite general guidelines, deciding on a reactor for Fenton and photo-Fenton processes required a manifold analysis. This work limits solar radiation to present an illustrative, non-exhaustive, comparison of three types of solar reactors Compound Parabolic Collectors (CPCs), Flat Collector Reactors (FPs), and Raceway Pond Reactors (RPR) referred to in the literature. According to Cabrera-Reina et al. [68], tubular photo reactors designed for efficient photon capture, as CPCs, have been used to treat high-polluted wastewater. Other reactors such as FP and RPR have also been tested and compared in terms of efficiency and cost to treat industrial wastewater by solar photo-Fenton. The reported properties and features are summarized in Table 6, and RPR is adopted for the next steps as the cost is prioritized as the main issue at this prototyping stage.

#### 3.3.1. Prototyping

**Figure 3:**
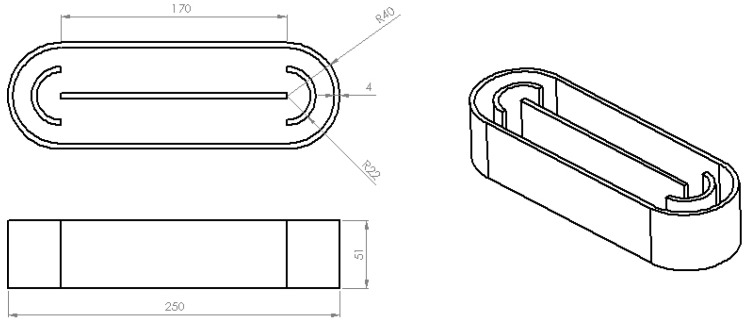
Scheme and dimensions (mm) of the lab-scale RPR.

Hence, the selected materials, PLA and Timberfill^®^, were used to 3D print lab-scale raceway ponds. According to the literature [68,69,70,71], the capacity of the prototype reactor was fixed at 0.5 L. A height of 51.0 mm was adopted assuming 50 mm for the optical path. The ratio length/width was set to 3, according to previous studies [47,69] recommending a value in the range 3–6. This led to a length of 250.0 mm and a width of 80.0 mm. Figure 3 gives the corresponding scheme.

**Figure 4:**
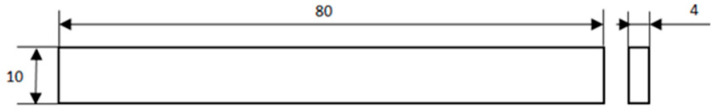
Shape and dimensions (mm) of the pieces used in the Flexural test (ASTM D6272).

Given the geometry of the reactor (RPR), materials need to be next assessed regarding the load they will bear while operating. This will be assessed by the minimum material thickness tolerating the maximum stress attained at any point of the reactor (Criterion 4.1). Certainly, sophisticated designs with variable thickness can be envisaged, but constant thickness is accepted as a reasonable condition to continue with the process of material selection. Hence, a Flexural test is proposed to evaluate the maximum stress resisted by the (pieces) materials, while the von Mises stress obtained using Finite Element Method (FEM) analysis is proposed to estimate the maximum stress in the reactor. The Flexural test was performed following the ASTM D6272 [72] standard. Five rectangular samples of each material (PLA and Timberfill^®^) were printed (Figure 4) using a Sigma printer by BCN3D (Barcelona, Spain).

The universal test machine Microtest has been used equipped with a 25-kN load cell, a 50-mm extensometer, a Spider, and a Microtest data acquisition system. The procedure used to measure and analyze the data is the same followed by Zandi et al. [47]. Hence, the maximum stress determined for Timberfill^®^ is 47.26 ± 0.86 MPa, which is much lower than that determined for PLA (109.50 ± 4.70 Mpa).

**Figure 5:**
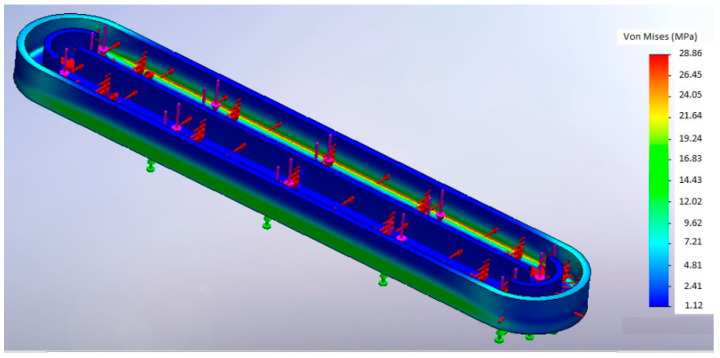
von Mises stress obtained from a Finite Element Method (FEM) simulation under a load given by 0.5 L of water.

SolidWorks has been used to run the Finite Element Method (FEM) analysis. Figure 5 illustrates a simulation of the von Mises stress under the load given by 0.5 L of water, while the results of the analysis for different thicknesses are given in Table 7. It also gives the maximum design stress assuming a 1.5 safety factor applied to the FEM estimated values.

The results in Table 7 reveal that PLA can bear the prototype maximum stress with any of the studied thicknesses (and probably with much lower values), while Timberfill^®^ requires a minimum thickness (45 mm according to the discrete values studied). Concerning Criterion 4.1, PLA is a better option. However, both materials are selected with a common thickness of 45 mm to continue with the comparative analysis. This is summarized in Table 8.

#### 3.3.2. Printing Parameters

FFF creates porosities in the printed pieces placed at the interface between the layers, so preventing liquid leakage in FFF-printed products is a challenge [69]. Usually, tuning these parameters is based on personal experience and know-how, but there is not enough comprehensive information to determine suitable manufacturing parameters [70,71]. Thus, printing parameters were selected from previous experiences of the research group [47,73], corresponding with the best mechanical properties. The printed parameters used are shown in Table 9.

**Figure 6:**
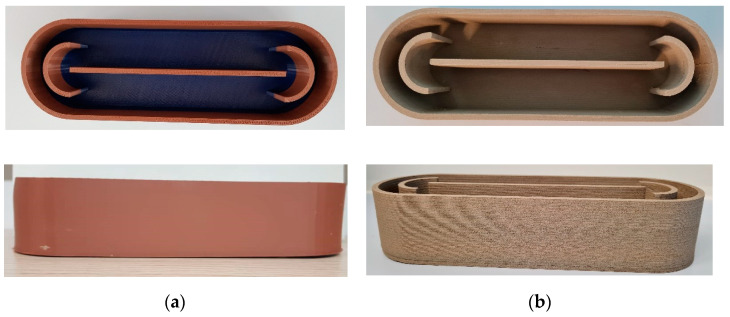
Picture of the 3D-printed race pond reactors (RPRs): PLA (**a**) and Timberfill^®^ (**b**).

Finally, the two Raceway Pond Reactors (RPRs) produced according to the printing specifications given in Table 9 are presented in Figure 6.

### 3.4. Prototype Testing and Selection

#### 3.4.1. Mechanical Test

Once printed, the raceway ponds reactors, Figure 6, need to be finally tested as a vessel aimed at containing a liquid solution. Hence, water tightness is paramount. It is well known that FFF creates porosities in the printed pieces placed at the interface between the layers, so preventing liquid leakage in FFF printed products is a challenge [69]. Increasing the nozzle diameter and the layer height are the usual printing strategies to achieve it. This was considered in Section 3.3.2 when the printing parameters were tuned. Next, the complete structure is put to test.

Thus, the reactors were filled with water. The criterion at this final stage (5.1) is not observing any leakage after 10 days. This lapse of time is deemed convenient, as regular use during the following months confirmed the vessel to be tightly sealed.

No leakage was detected during the given time in both printed reactors, which indicates that the selected design and the implementation (printing) parameters suffice to guarantee a reactor with robust cohesion between layers. This result is given in Table 10.

Table 10 summarizes the comparative assays carried out in this section. According to criteria 5, PLA and Timberfill resulted in good alternatives under the mechanical assessment section.

#### 3.4.2. Chemical Test

After assessing the performance as a container, the specific suitability of the different materials to be used as Fenton and photo-Fenton reactors is next tested and evaluated. While this may be to some extent redundant, the options are definitively confirmed in this final pilot test and compared to a reference reactor.

Such suitability was assessed in regard of two criteria:

- Criterion 6.1: Reaction viability; the capacity of the reactor to handle both Fenton and photo-Fenton reactions.
- Criterion 6.2: Material interference; the capability of the different reactors to significantly interfere with these reactions.

Accordingly, the performance of the 3D printed reactors is examined under conditions comparable to the Fenton and photo-Fenton treatment of an organic load. An aqueous caffeine solution (500 mL, 30 ± 0.5 mg·L^−1^) is used, and the following conditions are set: acidic pH (3 ± 0.2), 300 ± 10 mg·L^−1^ $H_{2}O_{2}$, 10 ± 0.1 mg·L^−1^ Fe (II). Experiments are conducted without light (Fenton process) and under UV light (photo-Fenton). The evolution of TOC, and $H_{2}O_{2}$ concentrations are monitored along the reaction time.

The same treatment is performed in 500 mL regular Pyrex^®^ Flask, which is intended as a control. Despite the different geometry, the Pyrex^®^ high resistance to chemical attack is assumed to be very informative as a reference.

**Figure 7:**
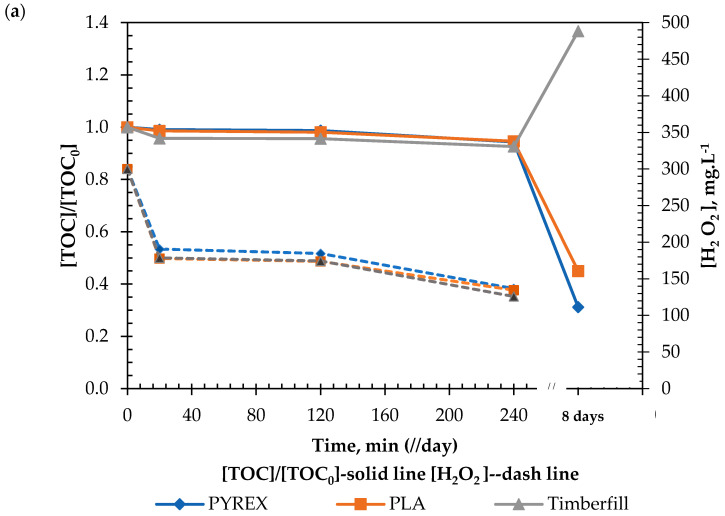

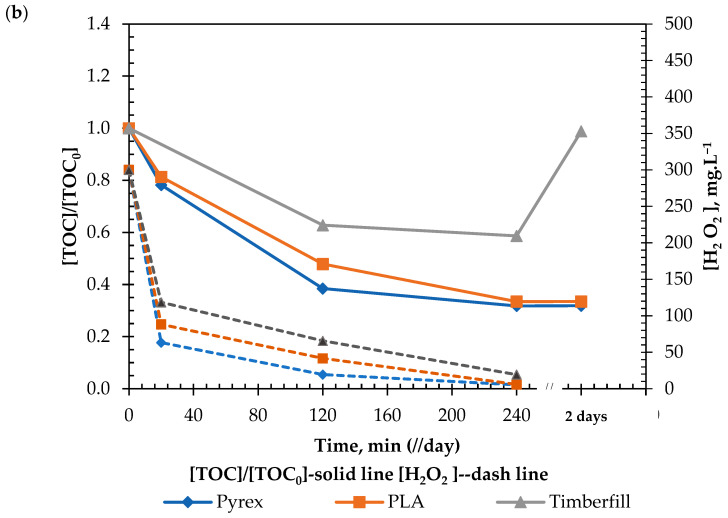
Evolution of TOC and $H_{2}O_{2}$ concentration (**a**) without UV and (**b**) with UV irradiation (30 ± 0.5 mg $L^{-1}$ caffeine solutions, ${\mathrm{TOC}}_{0}$ = 17.2 ± 1 mg $L^{-1}$ in PLA, Timberfill^®^, and Pyrex^®^ reactors. Experimental conditions: pH = 3 ± 0.2, $H_{2}O_{2}$ = 300 ± 10 mg $L^{-1}$.

The results of the different assays with synthetic water (Figure 7) readily confirmed that organic matter from the Timberfill^®^ reactor was contaminating the test solution in the long term, from which the interference of the material in the reaction is inferred.

Contrarywise, the same results indicated the promising capability of PLA to be used as a photo-Fenton reactor. The results from the PLA raceway pond were consistent with those obtained from the reference Pyrex^®^ reactor.

The experiments were repeated without light and under UV irradiation. These repetitions are consistent and show the parallel response of Fenton and photo-Fenton processes, although the presence of UV light speeds up the oxidation of the organic matter, as expected. In the short term (i.e., 240 min), PLA and Timberfill^®^ perform very similarly without light, although under UV light, Timberfill^®^ performs less efficiently than PLA. Thus, both can handle Fenton and photo-Fenton reactions (Criterion 6.1), but the lower efficiency of Timberfill^®^ indicates the interference of the material in the reaction and suggests the migration of organic matter from the material to the solution (Criterion 6.2). The PLA profile running quite parallel to the Pyrex^®^ profile also supports this idea, which is confirmed in the long term. After 8 days for the Fenton reaction and after only 2 days for the photo-Fenton reaction (faster, as expected) Timberfill^®^ is revealed as completely unsuitable in both cases due to the evident interaction of the material with the processes.

The results for the chemical tests performed with PLA and Timberfill^®^ and the conclusions drawn are provided in Table 11, which also shows the final assessment and decision.

## 4. Conclusions

Three-dimensional (3D printing is an enabling technology that allows producing low-cost reactor prototypes, and it also improve process design and validation in a reduced time. This study focused on 3D printing for the prototyping of Fenton and photo-Fenton reactors and systematically addressed the selection and testing of printing materials, as well as the design parameters to be considered in the printing process. In addition to the methodological approach, this work also contributes specific results regarding the problems detected in the preparation of 3D-printed reactors and their use for photo-Fenton processes.

Although metallic and ceramic materials were included in the analysis, they were readily discarded, as these options are currently unaffordable. Polymeric and composite materials are presently the only accessible choice. They were assessed and selected through systematically applying a set of criteria including biodegradability, and chemical, thermal, and mechanical resistance. In the first stage of the procedure, Acrylonitrile butadiene styrene (ABS), Polylactic acid (PLA), and Timberfill^®^ were identified as promising materials for prototyping photo-Fenton reactors.

Three-dimensional (3D) printing criteria were next analyzed by preparing reactor prototypes. Race pond reactors (RPR) were selected, and printed PLA and Timberfill^®^ lab-scale RPRs were tested for their capacity for holding the photo-Fenton reaction. Measurements of Total Organic Carbon (TOC) were produced and compared with those obtained for a standard Pyrex^®^ reactor under similar conditions (artificial UVA light, Fenton reagents, and contaminant).

The results of different assays confirm that no organic matter migrates from the container to the reaction solution when using the PLA reactor. Instead, the Timberfill^®^ container presents such migration, which was shown by TOC levels higher than should be expected. Thus, results confirm the promising capability of PLA to be used as a photo-Fenton reactor.

In addition to the systematic approach to material selection, the work also contributes to the identification of specific problems as leakage. The work addressed the issue accordingly by tuning the printing parameters and reporting the values that produce stronger cohesion between printed layers and ensures the reactor is watertight.

## Figures and Tables

**Table 1:** The most common sorts of materials used for additive manufacturing.

Metallic	Ceramic	Polymeric Base
Titanium	Alumina	Nylon
Aluminum	Zircon dioxide	Polycarbonate (PC)
Stainless steel	Hydroxyapatite	Polyvinyl alcohol (PVA)
Copper	Titanium oxide	Acrylonitrile butadiene styrene (ABS)
Inconel	Tri-calcium phosphate	Polylactic acid (PLA)
Gold/Platinum	Bio-glass	Composite PLA–wood fibers (Timberfill^®^)

**Table 2:** Pre-selection of the type of 3D-printing material based on chemical, mechanical, and economic criteria (Criteria #1).

Criteria #1	Criterion 1.1 Chemical Properties	Criterion 1.2 Mechanical Properties	Criterion 1.3 Manufacturing Cost	Decision
Metallic	Passed	Passed	Failed	Rejected
Ceramic	Passed	Passed	Failed	Rejected
Polymeric	Passed	Passed	Passed	Selected
Composite	Passed	Passed	Passed	Selected

**Table 3:** Pre-selection of 3D-printing polymeric and composite materials based on economic and operational criteria (Criteria #1).

Criteria #1	Criterion 1.4 Cost	Criterion 1.5 Heat Resistance	Criterion 1.6 Mechanical Strength	Criterion 1.7 Sustainability	Criterion 1.8 Water Solubility	Decision
Nylon	Failed	Failed	Passed	Non-biodegradable	Passed	Rejected
PC	Failed	Passed	Passed	Biodegradable	Passed	Rejected
PVA	Failed	Passed	Passed	Biodegradable	Failed	Rejected
ABS	Passed	Passed	Failed	Non-biodegradable	Passed	Selected
PLA	Passed	Passed	Passed	Biodegradable	Passed	Selected
Timberfill^®^	Passed	Passed	Failed	Biodegradable	Passed	Selected

**Table 4:** Mechanical comparison of PLA, Timberfill^®^, and ABS based on printability, stiffness, and required heated bed (Criteria #2).

Criteria #2	Criterion 2.1 Printability	Criterion 2.2 Stiffness	Criterion 2.3 Heated Bed	Decision
PLA	Passed	Passed	Not Required	Selected
Timberfill^®^	Passed	Passed	Not Required	Selected
ABS	Passed	Failed	Required	Rejected

**Table 5:** Chemical comparison of PLA, Timberfill^®^, and ABS based on chemical and thermal stability (Criteria #2).

Criteria #3	Criterion 3.1 & 3.2 Reaction Environment & Light Resistance	Criterion 3.3 Thermal Stability	Decision
PLA	Passed	Passed	Selected
Timberfill^®^	Passed	Passed	Selected
ABS	Failed	-	Rejected

**Table 6:** Comparison between different types of reactors (RPR, CPC, and FP) based on cost, efficiency, treatment capacity, and accumulated energy (Criteria #3).

	Cost	Efficiency (Common Polluted Wastewater)	Efficiency (High Polluted Wastewater)	Treatment Capacity	Accumulated Energy	Decision
RPR	Passed	Passed	Passed	Passed	Passed	Selected
CPC	Failed	Passed	Passed	Passed	Passed	Rejected
FP	Failed	Passed	Passed	Passed	Passed	Rejected

**Table 7:** Results of the Finite Element Method (FEM) analysis for different thicknesses (40–50 mm) of PLA and Timberfill^®^.

Thickness (mm)	*σ* von-Mises (MPa)	Maximum Design Stress (Safety Factor 1.5 Mpa)	Timberfill^®^ Maximum Stress (Mpa)	PLA Maximum Stress (Mpa)
40	38.4	57.5		
45	28.9	43.3	47.26 ± 0.86	109.50 ± 4.70
50	22.1	33.1		

**Table 8:** Comparison between PLA and Timberfill^®^ using maximum stress as a mechanical indicator (Criterion #4).

Criteria #4	Criterion 4.1 Maximum Stress	Decision
Timberfill^®^	Passed	Worst option
PLA	Passed	Best option

**Table 9:** Parameters used for 3D-printing the PLA and Timberfill^®^ reactors.

**Printing Parameters**			
**Parameter**	**Value**	**Parameter**	**Value**
Contour width	1.2 mm	Brim	5 mm
Solid upper layers width	1.2 mm	Overlap/contour intersection	15%
Solid lower layers width	1.2 mm	Support material	No
Extra contour	Required	Space between filaments	1.5 mm
Combine filling every	2 layers	Raft (base layer)	No
Flow ratios	1	Speed trips in vacuum	130 mm/s
**Extruder Parameters**			
Retraction length	2 mm	Extra length when reprinting	0 mm
Raise in Z	0 mm	Minimum distance for shrinkage	2 mm
Speed retraction	40 mm/s	Infill Pattern	Honeycomb
Layer height (mm)	0.2	Density (%)	75
Nozzle diameter (mm)	0.6 for PLA, 0.7 for Timberfill^®^		
Printing velocity (mm/s)	40 for PLA, 30 for Timberfill^®^		

**Table 10:** Testing and selection of PLA and Timberfill^®^ concerning leakage (Criterion #5).

Criteria #5	Criterion 5.1 Leakage	Decision
Timberfill^®^	Passed	Selected
PLA	Passed	Selected

**Table 11:** Final material assessment and selection between PLA and Timberfill^®^ (Criteria #6).

Criteria #6	Criterion 6.1 Reaction Viability	Criterion 6.2 Material Interference	Decision
PLA	Passed	Not observed	Selected
Timberfill^®^	Passed	Observed	Rejected

## Data Availability

Data is contained within the article.
